# Supplementary material for: RPLP1 restricts HIV-1 transcription by disrupting C/EBPβ binding to the LTR
Source: Nat Commun. 2024 Jun 21;15:5290. doi: 10.1038/s41467-024-49622-1 (PMC11192919; doi:10.1038/s41467-024-49622-1)
Supplement: Supplementary file 1 — Supplementary information [file 41467_2024_49622_MOESM1_ESM.pdf]

RPLP1 restricts HIV-1 transcription by disrupting C/EBPβ binding to the LTR

Weijing Yang[1-3], Hong Wang[1-3], Zhaolong Li[1-3], Lihua Zhang[4], Jianhui Liu[4], Frank Kirchhoff[5], Chen Huan[1-3]*, and Wenyan Zhang[1-3]*

[1]Department of Infectious Diseases, Infectious Diseases and Pathogen Biology Center, The First Hospital of Jilin University

[2]Institute of Virology and AIDS Research, The First Hospital of Jilin University

[3]Key Laboratory of Organ Regeneration and Transplantation of The Ministry of Education, The First Hospital of Jilin University

[4]State Key Laboratory of Medical Proteomics, Dalian Institute of Chemical Physics, Chinese Academy of Science

[5]Institute of Molecular Virology, Ulm University Medical Center, 89081 Ulm, Germany

*Corresponding authors:

Wenyan Zhang, E-mail: zhangwenyan@jlu.edu.cn, and Chen Huan, E-mail: lmsxsml@jlu.edu.cn

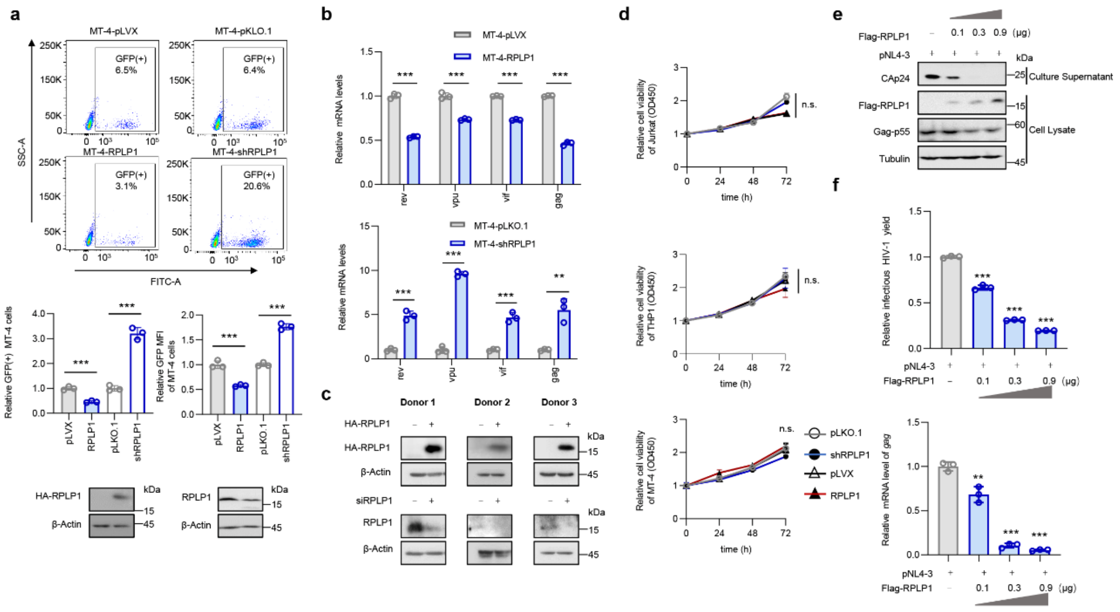

Supplementary Fig. 1 (Related to Fig. 1). RPLP1 inhibits HIV-1 replication.

a-**b** RPLP1 inhibits HIV-1 replication in MT-4 cells. **a** MT-4 cells with altered RPLP1 levels (lower) were infected with HIV-1 NL4-3-EGFP virus, and the percentage of GFP-positive cells was measured by flow cytometry at 48 hours post-infection (upper). The ratio of GFP-positive cells and relative MFI compared to control cells was calculated (middle). **b** Quantification of HIV-1 proteins mRNA levels in MT4 cells in (**a**). **c** Immunoblotting analyses of RPLP1 levels in primary CD4+ T cells nucleofected with HA-RPLP1 or siRNA against RPLP1 in Fig. 1 (**d**). **d** Alternation of RPLP1 didn't affect viability of T cells. **e**-**f** RPLP1 inhibits HIV-1 replication in a dose-dependent manner. HEK293T cells were co-transfected with pNL4-3 viral vector and increasing amounts of Flag-RPLP1. Cells and culture supernatant were harvested at 48 h post transfection for immunoblotting analysis (**e**). Infectious virus yield was quantified using the TZM-bl reporter cell infectivity assay, and the mRNA level of HIV-1 *gag* was measured using RT-qPCR (**f**). Immunoblots in (**a**, **c** and **e**) are representative of three independent experiments. Quantification in (**a**, **b**, **d** and **f**) was shown as means ± SDs from three independent experiments. *P* values were calculated by the two-tailed student's *t*-test. $^{**}P < 0.01$, $^{***}P < 0.001$, n.s. denotes no significance. Source data are provided as a Source Data file.

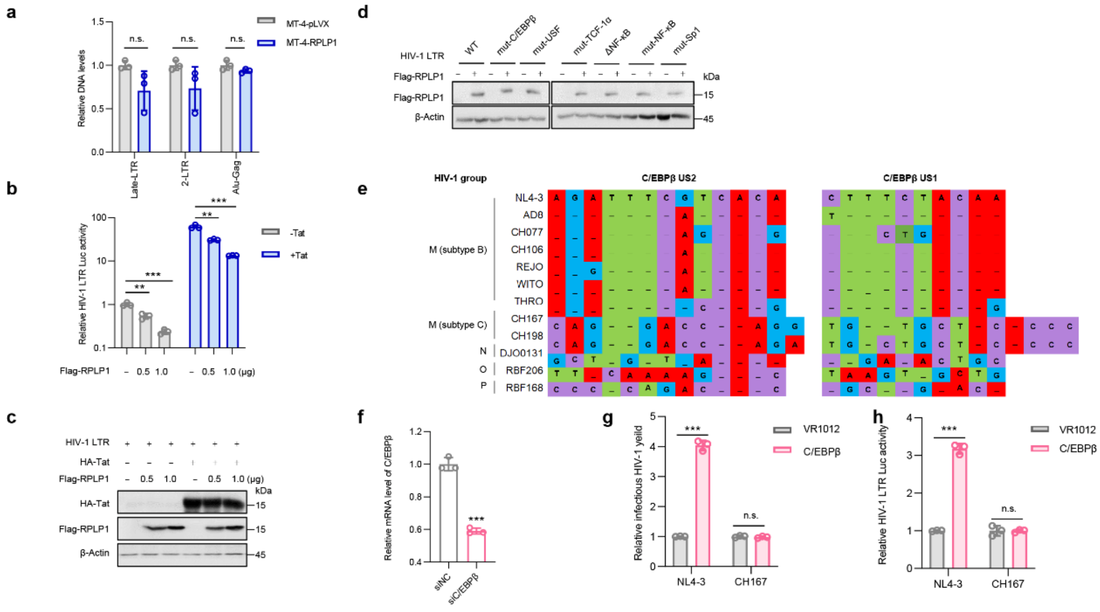

Supplementary Fig. 2 (Related to Fig. 2). RPLP1 inhibits HIV-1 replication by suppressing viral LTR activity.

a HIV-1 reverse transcription, nuclear entry and integration were not affected by RPLP1. The DNA from control and MT-4-RPLP1 cells infected with HIV-1 NL4-3 virus was extracted 48 hours after infection. Levels of late-RT, 2-LTR, and two-step Alu were quantified using PCR, with values from control cells set as 1. **b-c** RPLP1 suppresses HIV-1 LTR activity in a Tat-independent manner. HEK293T cells were transfected with indicated plasmids. Cells were collected 48 hours post-transfection and LTR activity was measured (**b**), and protein levels were determined by immunoblotting (**c**). **d** Immunoblotting analysis of cells in Fig. 2 (**b**). **e** Multiple sequence alignment of various HIV-1 group M (subtype B and C), group N, O and P LTR referenced to the C/EBPβ binding sites. **f** RT-qPCR analysis of C/EBPβ levels of cells in Fig. 2 (**e**). **g** C/EBPβ promoted HIV-1 production of subtype B (NL4-3) but not subtype C (CH167). HIV-1 constructs of NL4-3 or CH167 were transfected into HEK293T cells with or without Myc-C/EBPβ, and infectious virus yield was quantified using the TZM-bl reporter cell infectivity assay. **h** C/EBPβ enhances HIV-1 LTR activity of subtype B (NL4-3), but not subtype C (CH167)**.** HEK293T cells were transfected with indicated plasmids for 48 hours and LTR activity was measured. Immunoblots in (**c** and **d**) are representative of three independent experiments. Quantification in (**a**, **b** and **f-h**) was shown as means ± SDs from three independent experiments. *P* values were calculated by the two-tailed student's *t*-test. **P* < 0.05, ***P* < 0.01, ****P* < 0.001, n.s. denotes no significance. Source data are provided as a Source Data file.

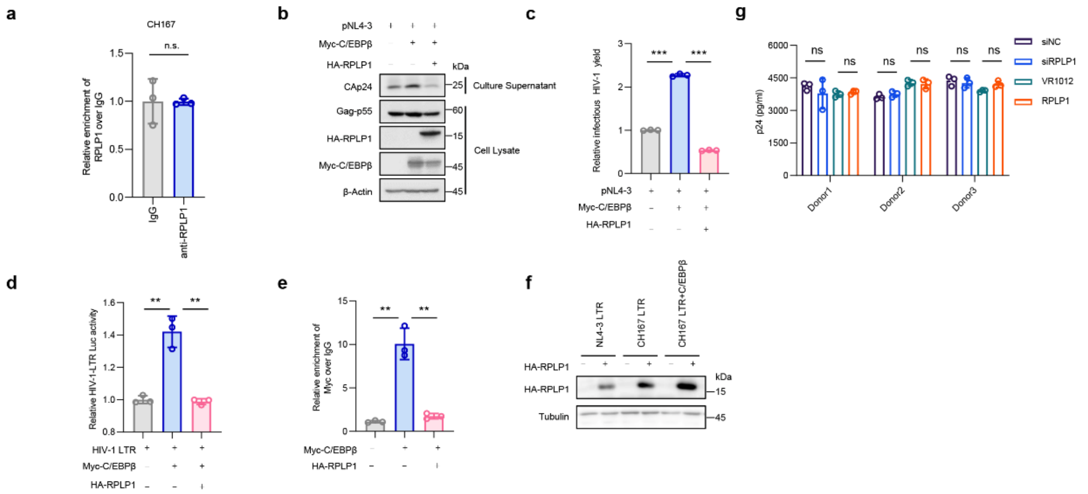

Supplementary Fig. 3 (Related to Fig. 3). RPLP1 competes with the transcription factor C/EBPβ to bind HIV-1 LTR.

a RPLP1 was not recruited to the HIV-1 CH167 LTR spanning the C/EBPβ binding sites. Chromatin from HIV-1 CH167-infected Jurkat cells was isolated and immunoprecipitated with anti-RPLP1 antibody or IgG as a control, followed by RT-qPCR analysis using primers targeting C/EBPβ binding sites in the LTR. **b-c** HIV-1 promoting activity of C/EBPβ was impaired by RPLP1. HEK293T cells were transfected with pNL4-3 viral vector along with Myc-C/EBPβ, with or without HA-RPLP1. Cells and culture supernatant were collected at 48 hours post transfection for immunoblotting analysis (**b**), and infectious virus yield was quantified using the TZM-bl reporter cell infectivity assay (**c**). **d** RPLP1 suppressed HIV-1 LTR activity stimulated by C/EBPβ. HEK293T cells were transfected with indicated plasmids and LTR activity was measured as48 hours post transfection. **e** RPLP1 impaired the recruitment of C/EBPβ to the HIV-1 LTR. HEK293T cells were transfected with Myc-CEBPβ and/or HA-RPLP1, then infected with HIV-1 NL4-3 for 2 days before ChIP assays using anti-Myc antibody. Then, RT-qPCR analysis was performed using primers targeting C/EBPβ binding sites in the LTR. **f** Immunoblotting analysis of cells in Fig. 3 (**f**). **g** RPLP1 does not inhibit HIV-1 subtype C replication in primary CD4+ T cells. CD4+ T cells from HIV-negative participants (n=3 donors) were nucleofected with HA-RPLP1 or siRNA against RPLP1, then infected with HIV-1 CH167. HIV-1 yield in supernatant was measured with p24 ELISA assay. Immunoblots in (**b** and **f**) are representative of three independent experiments. Quantification in (**a**, **c-e**, and **g**) was shown as means ± SDs from three independent experiments. *P* values were calculated by the two-tailed student's *t*-test. ***P* < 0.01, ****P* < 0.001, n.s. denotes no significance. Source data are provided as a Source Data file.

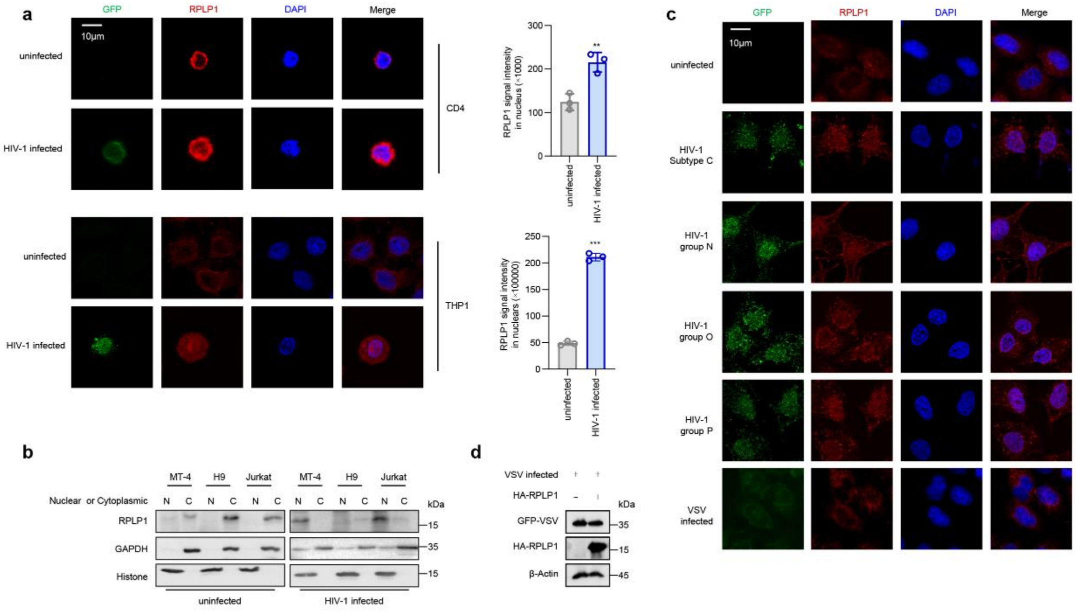

Supplementary Fig. 4 (Related to Fig. 4). HIV-1 infection induces cytoplasm-to-nucleus translocation of RPLP1.

a-b HIV-1 infection induced cytoplasm-to-nucleus translocation of RPLP1. **a** CD4+ T cells isolated from HIV-negative study participants (n=3) or THP1 cells were infected with HIV-1 NL4-3-EGFP virus for 48 h, then the cells were detected by immunofluorescence assay, and images were captured with a Zeiss LZM710 confocal microscope. Scale bars, 10 μm (leftl). The fluorescence intensity of RPLP1 in nucleus were calculated using ImageJ (right). **b** MT-4, H9 and Jurkat cells were infected with NL4-3 virus for 48 h, and then the nuclear-cytoplasm separation assay was performed. N, nuclear; C, cytoplasm. **c** Various subtypes/groups of HIV-1, but not VSV infection induced cytoplasm-to-nucleus translocation of RPLP1.HeLa cells were infected with various subtypes/groups of HIV-1 for 48 h, then the cells were detected by immunofluorescence assay, and images were captured with a Zeiss LZM710 confocal microscope. **d** RPLP1 did not affect VSV replication. HEK293T cells were transfected with control vector or HA-RPLP1 and infected with GFP pseudo-typed VSV at 8 h post transfection. Forty hours later, the cells were collected, and protein levels in cells were determined by immunoblotting. Immunoblots in (**b** and **d**) and immunofluorescence images in (**a** and **c**) are representative of three independent experiments. Quantification in (**a**) was shown as means ± SDs from three independent experiments. *P* values were calculated by the two-tailed student's *t*-test. ***P* < 0.01, ****P* < 0.001. Source data are provided as a Source Data file.

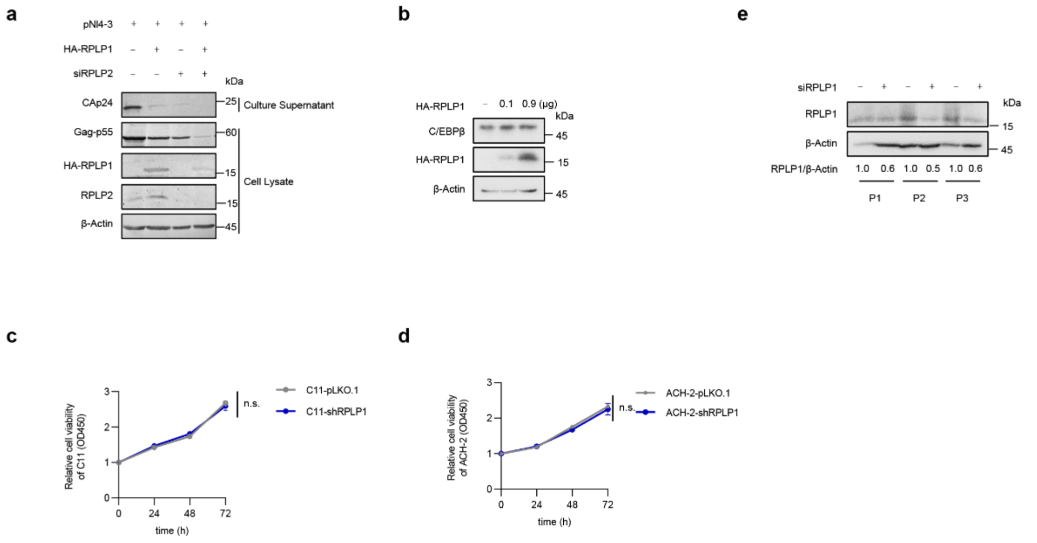

Supplementary Fig. 5. The RPLP1 counteraction on HIV-1 is independent with its protein translation function or its impact on C/EBPβ levels.

a The protein translation function was not involved in the anti-HIV-1 effect induced by RPLP1. Control or RPLP2 silencing HEK293T cells were co-transfected with HIV-1 constructs plus HA-RPLP1, and the cells and culture supernatant were harvested for immunoblotting with the indicated antibodies at 48 h post transfection. **b** C/EBPβ level was not affect by over-expression of RPLP1. HEK293T cells were transfected with indicated amounts of HA-RPLP1, and the endogenous C/EBPβ level was examined with immunoblotting at 48 h post transfection. **c** Silencing of RPLP1 didn't affect viability of C11 cells. The cell viability and growth of the control and RPLP1 silencing C11 cells was detected using the CCK8 assay, and the corresponding values of the control cells at 0 h were set as 1. **d** Silencing of RPLP1 didn't affect viability of ACH-2 cells. The cell viability and growth of the control and RPLP1 silencing ACH-2 cells was detected using the CCK8 assay, and the corresponding values of the control cells at 0 h were set as 1. **e** Immunoblotting analysis of primary CD4+ T cells in Fig. 6 (**i**), P1-P3 demoted three participants. Immunoblots in (**a**, **b** and **e**) are representative of three independent experiments. Quantification in (**c** and **d**) was shown as means ± SDs from three independent experiments. The densitometric analysis of protein levels in (**e**) is shown as the mean value (n = 2) relative to β-Actin. *P* values were calculated by the two-tailed student's *t*-test. n.s. denotes no significance. Source data are provided as a Source Data file.

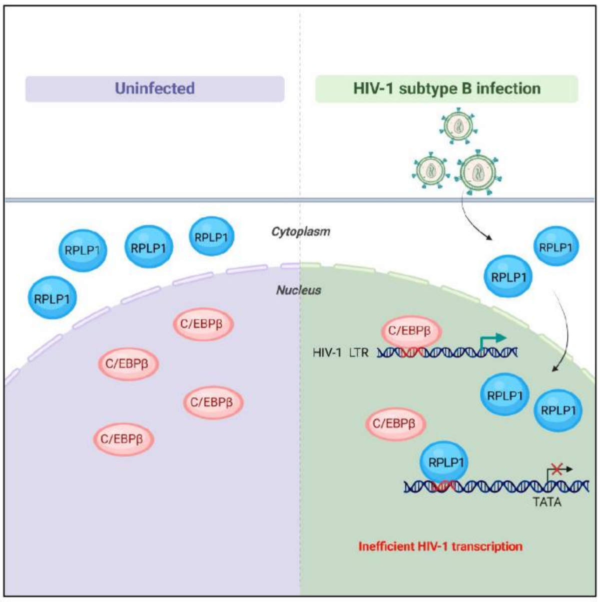

Supplementary Fig. 6 Proposed inhibition mechanism of RPLP1 on the replication of HIV-1 subtype B.

Normally, RPLP1 locates in cytoplasm, while the HIV-promoting transcription factor C/EBPβ locates in nucleus (left panel). Upon HIV-1 subtype B infection, cytoplasm-to-nucleus translocation of RPLP1 occurred and RPLP1 in the nucleus competed with C/EBPβ to bind the C/EBPβ sites of HIV-1 LTR, thus inducing transcription inhibition on HIV-1 by disrupting the HIV-1 LTR- C/EBPβ interaction (right panel). Image is generated using BioRender (http://biorender.com/).

Supplementary Table 1 Characteristics of LTNPs and RPs enrolled for proteomic profiling analysis.

Characteristics[a]	Long-term non-progressors (LTNP, n=3)	Regular progressors (RP, n=2)	*P* value
Age (yrs)	58 ± 6	57 ± 10	NS
HIV-1 subtype	B	B	/
antiretroviral therapy	-	+	/
Duration of HIV infection (yrs)	18 ± 2	18 ± 1	NS
CD4+ T counts (cells/mm^3)	833 ± 152	340 ± 63	0.019
Viral load (HIV RNA copies/ml plasma)	＜50	＞700	/

a Data are shown as means ± SDs. *P* values were calculated by the two-tailed student's *t*-test. n.s. denotes no significance.

Supplementary Table 2 Characteristics of cART treated HIV-1-infected individuals in this study.

Characteristic	cART treated HIV-1-infected individuals
No. of individuals	3
Sex	Male
Age（yrs）	36 ± 13
CD4+ T counts (cells/mm^3)	972 ± 151
Viral load (HIV RNA copies/ml plasma)	<50
Antiretroviral therapy	yes
Duration of antiretroviral therapy	＞6 months

Supplementary Table 3 Primers used for plasmid construction.

Primer name	Primer direction	Sequence (5'-3')
Flag-RPLP-EcoRI-F	Forward	CGGAATTCATGGCCTCTGTCTCCGAGG
Flag-RPLP-BamHI-R	Reverse	CGGGATCCTTACTTATCGTCGTCATCCTTGTAATCGTCAAAAAGACCAAAGCC
HA-RPLP1- F	Forward	GGCTTTGGTCTTTTTACCCATACGATGTTCCAGATTACGCTTGAGGATCCAGATC
HA-RPLP1- R	Reverse	AAAAAAGACCAAAGCCCATGTCATCA
HA-RPLP1-Δα1-F	Forward	CACCATGGCCTCACTGCACGACGATG
HA-RPLP1-Δα1-R	Reverse	GTGAGGCCATGGTGTCGACGACG
HA-RPLP1-Δα2-F	Forward	GGTGACAGTCACGGGTGTAAATGTTGAG
HA-RPLP1-Δα2-R	Reverse	CATCCTCCGTGACTGTCACCTCATCGTCG
HA-RPLP1-Δα3-F	Forward	GGTGTAAATGTTGAGCTGGCCAACGTC
HA-RPLP1-Δα3-R	Reverse	GCTCAACATTTACACCGGCTGCTTTAATG
HA-RPLP1-Δα4-F	Forward	CCAACGTCAACATTAATGTAGGGGCCG
HA-RPLP1-Δα4-R	Reverse	TAATGTTGACGTTGGCCAGGGCCTTTG
pLVX-RPLP1-BamHI-F	Forward	CGGGATCCGCCTCAGTCTCCGAGCTC
pLVX-RPLP1-XhoI-R	Reverse	GCTCGAGCCAAAAAGACCAAAGCCCATGTC
shRPLP1-F	Forward	CCGGGGAGAAGAAAGTGGAAGCACTCGAGTGCTTCCACTTTCTTCTCCTTTTTG
shRPLP1-R	Reverse	AATTCAAAAAGGAGAAGAAAGTGGAAGCACTCGAGTGCTTCCACTTTCTTCTCC
HIV1-LTR-mutNF-κB/NFAT I-F	Forward	TCGAGCTTTCTACAACTCACTTTCCGCTGGG
HIV1-LTR-mutNF-κB/NFAT I-R	Reverse	GAGTTGTAGAAAGCTCGATGTCAGCAGT
HIV1-LTR-mutNF-κB/NFAT II-F	Forward	GGACTTTCCGCTGCTCACTTTCCAGGGAGG
HIV1-LTR-mutNF-κB/NFAT II-F	Reverse	GAGCAGCGGAAAGTCCCTTGTAGAAAG
HIV1-LTR-mutC/EBPβ I-F	Forward	GCCTCCTAGCATTTAGTCACATGGCCC
HIV1-LTR-mutC/EBPβ I-R	Reverse	TAAATGCTAGGAGGCTGTCAAACTT
HIV1-LTR- mutC/EBPβ II-F	Forward	TGCTGACATCGAGAGCTGTACAAGGGACTTTC
HIV1-LTR- mutC/EBPβ II-R	Reverse	CAGCTCTCGATGTCAGCAGTCTTTGTAG
HIV1-LTR-mutSP1 I-F	Forward	GGCGGGACTGGTTAGTGGCGAGCCC
HIV1-LTR-mutSP1 I-R	Reverse	AACCAGTCCCGCCCAGGCCACACC
HIV1-LTR-mutSP1 II-F	Forward	AGGTGTGGCCTGTTCGGGACTGGGGA

HIV1-LTR-mutSP1 II-R	Reverse	AACAGGCCACACCTCCCTGGAAA
HIV1-LTR-mutSP1 III-F	Forward	GACTTTCCAGGGATTTGTGGCCTGGGC
HIV1-LTR-mutSP1 III-R	Reverse	AATCCCTGGAAAGTCCCCAGCGGA
HIV1-LTR-mutTCF-1α-F	Forward	GCATCCGGAGTACGAATTCGACTGCTGACATC
HIV1-LTR-mutTCF-1α-R	Reverse	GAATTCGTACTCCGGATGCAGCTCTCGGG
HIV1-LTR-mutUSF-F	Forward	CTCCTAGCATTTCGTGAATTCGCCCGAGAGCTG
HIV1-LTR-mutUSF-R	Reverse	CACGAAATGCTAGGAGGCTGTCAAAC
CH167-LTR-N6-1-F	Forward	TATTACAAAGACTGCCTTTCTACAAGGGACTTTCCGCT
CH167-LTR-N6-1-R	Reverse	CGCAGTCTTTGTAATACTCCGGATG
CH167-LTR-N6-2-F	Forward	GACAGTGAACTAGCATTTCGTCACATAGCCCGC
CH167-LTR-N6-2-R	Reverse	GAGTTCACTGTCATACTTCCA

Supplementary Table 4 Primers used for qRT-PCR

Primer name	Primer direction	Sequence (5'-3')
GAPDH-RT-F	Forward	TGCACCACCAACTGCTTAGC
GAPDH-RT-R	Reverse	GGCATGGACTGTGGTCATGAG
HIV-Gag-RT-F	Forward	GTGTGGAAAATCTCTAGCAGTGG
HIV-Gag-RT-R	Reverse	CGCTCTCGCACCCATCTC
HIV-Vif-RT-F	Forward	AGTTCAGAAGTACACATCCCA
HIV-Vif-RT-R	Reverse	GGGTCTACTTGTGTGCTATAT
HIV-Vpu-RT-F	Forward	ATAGCAATAGTTGTGTGGTCC
HIV-Vpu-RT-R	Reverse	CCATCTCCACAAGTGCTGAT
HIV-Rev-RT-F	Forward	AGACTCATCAAGCTTCTCTATC
HIV-Rev-RT-R	Reverse	TTCCACAATCCTCGTTACAATC
Late-LTR-RT-F	Forward	TGTGTGCCCGTCTGTTGTGT
Late-LTR-RT-R	Reverse	GAGTCCTGCGTCGAGAGATC
2-LTR-RT-F	Forward	AACTAGGGAACCCACTGCTTAAG
2-LTR-RT-R	Reverse	TCCACAGATCAAGGATATCTTGTC
first-Alu-F	Forward	AGCCTCCCGAGTAGCTGGGA
first-Alu-R	Reverse	TTACAGGCATGAGCCACCG
first-gag-R	Reverse	CAATATCATACGCCGAGAGTGCGCGCTTCAGCAAG
second-LTR-F	Forward	TTGTTACACCCTATGAGCCAGC
second-tag-R	Reverse	CAATATCATACGCCGAGAGTGC
C/EBPβ-RT-F	Forward	AGAAGACCGTGGACAAGCACAG
C/EBPβ-RT-R	Reverse	CTCCAGGACCTTGTGCTGCGT
ChIP-F1	Forward	GAGCTGCATCCGGAGTAC
ChIP-R1	Reverse	CCAGTCCCGCCCAGGC
ChIP-F2	Forward	GATGGTGCTTCAAGTTAGTAC
ChIP-R2	Reverse	CTAGGAGGCTGTCAAACTTC
