# Supplementary material for: RPLP1 restricts HIV-1 transcription by disrupting C/EBPβ binding to the LTR
Source: Nat Commun. 2024 Jun 21;15:5290. doi: 10.1038/s41467-024-49622-1 (PMC11192919; doi:10.1038/s41467-024-49622-1)
Supplement: Supplementary file 3 — reporting-summary [file 41467_2024_49622_MOESM3_ESM.pdf]

nature portfolio

Corresponding author(s): Wenyan Zhang

Last updated by author(s): Jun 5, 2024

Reporting Summary

Nature Portfolio wishes to improve the reproducibility of the work that we publish. This form provides structure for consistency and transparency in reporting. For further information on Nature Portfolio policies, see our Editorial Policies and the Editorial Policy Checklist.

Statistics

For all statistical analyses, confirm that the following items are present in the figure legend, table legend, main text, or Methods section.

n/a	Confirmed	
☐	☒	The exact sample size (*n*) for each experimental group/condition, given as a discrete number and unit of measurement
☐	☒	A statement on whether measurements were taken from distinct samples or whether the same sample was measured repeatedly
☐	☒	The statistical test(s) used AND whether they are one- or two-sided<br>*Only common tests should be described solely by name; describe more complex techniques in the Methods section.*
☐	☒	A description of all covariates tested
☒	☐	A description of any assumptions or corrections, such as tests of normality and adjustment for multiple comparisons
☐	☒	A full description of the statistical parameters including central tendency (e.g. means) or other basic estimates (e.g. regression coefficient) AND variation (e.g. standard deviation) or associated estimates of uncertainty (e.g. confidence intervals)
☐	☒	For null hypothesis testing, the test statistic (e.g. *F*, *t*, *r*) with confidence intervals, effect sizes, degrees of freedom and *P* value noted<br>*Give P values as exact values whenever suitable.*
☐	☒	For Bayesian analysis, information on the choice of priors and Markov chain Monte Carlo settings
☒	☐	For hierarchical and complex designs, identification of the appropriate level for tests and full reporting of outcomes
☒	☐	Estimates of effect sizes (e.g. Cohen's *d*, Pearson's *r*), indicating how they were calculated

Our web collection on statistics for biologists contains articles on many of the points above.

Software and code

Policy information about availability of computer code

Data collection	Mass spectrometric data were collected on an Orbitrap Fusion Lumos Tribrid mass spectrometer.<br>Olympus FV 3000 confocal imaging system was used to obtain confocal images.<br>Roche 480 instrument Real-time PCR system was used to collect quantitative PCR data.<br>BD FACSverse cytometer driven by FACSuite 1.0.3 was used to collect folw cytometry data.<br>Azure Biosystem was used to collect western blot data.<br>GloMax 20/20 luminometer (Promega) was used to collect luciferase data.
Data analysis	Data were analyzed by GraphPad Prism 8.2 Software.<br>FlowJo7.6.1 was utilized to analyze flow cytometry data.<br>Image J was used to quantify western blots and confocal images.<br>The linear models for microarray data (limma) was used to perform statistical analysis on mass spectrometry data, and the R code used in this study with descriptions in detail was provided as a supplement.

For manuscripts utilizing custom algorithms or software that are central to the research but not yet described in published literature, software must be made available to editors and reviewers. We strongly encourage code deposition in a community repository (e.g. GitHub). See the Nature Portfolio guidelines for submitting code & software for further information.

Data

Policy information about availability of data

All manuscripts must include a data availability statement. This statement should provide the following information, where applicable:

- Accession codes, unique identifiers, or web links for publicly available datasets
- A description of any restrictions on data availability
- For clinical datasets or third party data, please ensure that the statement adheres to our policy

Mass spectrometry data are available in iProX (PXD050294). Please verify this data using the provided link (https://www.iprox.cn//page/project.html?id=IPX0008310000). Code are available in github. Please verify this data using the provided link (https://github.com/wuruihongJilin/RPLP1study.git). All other data are available in the main text or in the supplemental information. Source data are provided with this paper.

Research involving human participants, their data, or biological material

Policy information about studies with human participants or human data. See also policy information about sex, gender (identity/presentation), and sexual orientation and race, ethnicity and racism.

Reporting on sex and gender	No sex- or gender-based designs or analyses were performed.
Reporting on race, ethnicity, or other socially relevant groupings	Study participants are Chinese.
Population characteristics	Study participants characteristics are provided in Extended Data Table 1 and 2
Recruitment	Participants were enrolled into the study HIV-1 infected individuals who remain asymptomatic with normal and stable CD4+ T cell counts for more than 10 years after diagnosis in the absence of combination antiretroviral therapy (cART) and more than 17 years and lack of other viral infections.
Ethics oversight	This is a study approved by the the ethics committee of the First Hospital of Jilin University (license number 22K086-001) . All participants were over 18 years old and provided written informed consent.

Note that full information on the approval of the study protocol must also be provided in the manuscript.

Field-specific reporting

Please select the one below that is the best fit for your research. If you are not sure, read the appropriate sections before making your selection.

[x] Life sciences [ ] Behavioural & social sciences [ ] Ecological, evolutionary & environmental sciences

For a reference copy of the document with all sections, see nature.com/documents/nr-reporting-summary-flat.pdf

Life sciences study design

All studies must disclose on these points even when the disclosure is negative.

Sample size	The sample sizes were chosen on the basis of previous experience in accordance to the standards in the field. Experiments were performed at least 3 times to confirm reproducibility. Sample size information was described in Methods and Figure legends.
Data exclusions	No data are excluded.
Replication	The data derived from cell lines were obtained from three independent experiment. The data derived primary cells isolated from healthy donors were generated from multiple independent experiments with independent donors (sample size, n was clearly described in Figure legends as well as Methods). All experiments are reproduced to reliably support conclusions stated in the manuscript and assay reproducibility is described in Figure legends.
Randomization	Samples are all randomly allocated to different experimental groups in this study. No specific randomization protocol has been used.
Blinding	Blinding was not performed and is not relevant in this study.

Reporting for specific materials, systems and methods

We require information from authors about some types of materials, experimental systems and methods used in many studies. Here, indicate whether each material, system or method listed is relevant to your study. If you are not sure if a list item applies to your research, read the appropriate section before selecting a response.

Materials & experimental systems

n/a	Involved in the study
☐	☒ Antibodies
☐	☒ Eukaryotic cell lines
☒	☐ Palaeontology and archaeology
☒	☐ Animals and other organisms
☒	☐ Clinical data
☒	☐ Dual use research of concern
☒	☐ Plants

Methods

n/a	Involved in the study
☒	☐ ChIP-seq
☐	☒ Flow cytometry
☒	☐ MRI-based neuroimaging

Antibodies

Antibodies used

The antibodies used in this study are listed as follows: Rabbit polyclonal anti-HA(SG77) (#715500, Thermo), Mouse monoclonal anti-Myc (clone 4A6) (#05-724, Millipore, Billerica, MA, USA), Mouse monoclonal anti-Flag (M2) (#F1804, Sigma, St. Louis, MO, USA), Mouse monoclonal anti-β-Actin (#A00702, GenScript Corporation, PISCATAWAY, NJ, USA), Rabbit polyclonal anti-Histone H3 (#A01502, GenScript Corporation), Mouse monoclonal anti-p24 (catalog no. 1513; AIDS Research and Reference Reagents Program [ARRRP], USA), Mouse monoclonal anti-β-Tubulin (MG7) (#RM2003, Ray Antibody Biotech, Beijing, China), Mouse monoclonal anti-GAPDH(MC4) (#RM2002, Ray Antibody Biotech), Rabbit polyclonal anti-RPLP1 (#21636-1-AP, Proteintech, Rosemont, IL, USA), Rabbit polyclonal anti-RPLP2 (ab154958, Abcam, Cambridge, UK), Rabbit polyclonal anti-C/EBPβ (D155298-0025, BBI, China), Peroxidase AffiniPure Goat Anti-Mouse IgG (H+L), (#115-035-062, Jackson, West Grove, PA, USA), Peroxidase AffiniPure Goat Anti-Rabbit IgG (H+L), (#111-035-045, Jackson).

Validation

All antibodies used in this study are commercial and well-established in the field. Validation data are provided for each antibody on the manufacturers' websites with antibody profiles and citations: Rabbit polyclonal anti-HA(SG77) (#715500, Thermo, 1:250), Mouse monoclonal anti-Myc (clone 4A6) (#05-724, Millipore, Billerica, MA, USA, 1:1000), Mouse monoclonal anti-Flag (M2) (#F1804, Sigma, St. Louis, MO, USA, 1:1000), Mouse monoclonal anti-β-Actin (#A00702, GenScript Corporation, PISCATAWAY, NJ, USA, 1:2000), Rabbit polyclonal anti-Histone H3 (#A01502, GenScript Corporation, 1:1000), Mouse monoclonal anti-p24 (catalog no. 1513; AIDS Research and Reference Reagents Program [ARRRP], USA, 1:500), Mouse monoclonal anti-β-Tubulin (MG7) (#RM2003, Ray Antibody Biotech, Beijing, China, 1:1000), Mouse monoclonal anti-GAPDH(MC4) (#RM2002, Ray Antibody Biotech, 1:1000), Rabbit polyclonal anti-RPLP1 (#21636-1-AP, Proteintech, Rosemont, IL, USA, 1:500), Rabbit polyclonal anti-RPLP2 (ab154958, Abcam, Cambridge, UK, 1:1000), Rabbit polyclonal anti-C/EBPβ (D155298-0025, BBI, China, 1:1000), Peroxidase AffiniPure Goat Anti-Mouse IgG (H+L), (#115-035-062, Jackson, West Grove, PA, USA, 1:10000), Peroxidase AffiniPure Goat Anti-Rabbit IgG (H+L), (#111-035-045, Jackson, 1:10000).

Eukaryotic cell lines

Policy information about cell lines and Sex and Gender in Research

Cell line source(s)

Human embryonic kidney 293T (HEK293T) (catalog no. CRL-11268), HeLa (catalog no. CCL-2) and TZM-bl (catalog no. PTA-5659) cells were obtained from American Type Culture Collection (ATCC; Manassas, VA, USA) and cultured in Dulbecco's modified Eagle's medium (11995065, Thermo) supplemented with 10% fetal bovine serum (FBS; ST30-3302, PAN Seratech, Aidenbach, Germany). The HIV-1 latent C11 cell line was a gift from H. Z. Zhu (The College of Life Science, Fudan University). ACH-2 (catalog no. 349) and MT-4 (catalog no. 120) cells were obtained from the AIDS Research and Reference Reagents Program, Division of AIDS, NIAID, NIH. Jurkat (ATCC catalog no. TIB-152), H9 (ATCC catalog no. HTB-176), THP1 (ATCC catalog no. TIB-202)), C11 and MT-4 cells were cultured in RPMI 1640 medium supplemented with 10% FBS and Penicillin-streptomycin Solution (03-031-1B, Biological Industries, Israel).

Authentication

All cell lines have not been authenticated

Mycoplasma contamination

We tested all of cell lines and no cell line found with any mycoplasma contamination.

Commonly misidentified lines
(See ICLAC register)

No commonly misidentified cell lines were used.

Plants

Seed stocks

Report on the source of all seed stocks or other plant material used. If applicable, state the seed stock centre and catalogue number. If plant specimens were collected from the field, describe the collection location, date and sampling procedures.

Novel plant genotypes

Describe the methods by which all novel plant genotypes were produced. This includes those generated by transgenic approaches, gene editing, chemical/radiation-based mutagenesis and hybridization. For transgenic lines, describe the transformation method, the number of independent lines analyzed and the generation upon which experiments were performed. For gene-edited lines, describe the editor used, the endogenous sequence targeted for editing, the targeting guide RNA sequence (if applicable) and how the editor was applied.

Authentication

Describe any authentication procedures for each seed stock used or novel genotype generated. Describe any experiments used to assess the effect of a mutation and, where applicable, how potential secondary effects (e.g. second site T-DNA insertions, mosiacism, off-target gene editing) were examined.

Flow Cytometry

Plots

Confirm that:

- [x] The axis labels state the marker and fluorochrome used (e.g. CD4-FITC).
- [x] The axis scales are clearly visible. Include numbers along axes only for bottom left plot of group (a 'group' is an analysis of identical markers).
- [x] All plots are contour plots with outliers or pseudocolor plots.
- [x] A numerical value for number of cells or percentage (with statistics) is provided.

Methodology

Sample preparation	MT4, Jurkat, THP1 cell lines were infected with HIV-1 virus. C11 cell lines were treated with PMA and SAHA.
Instrument	FACS Canto flow cytometer (BD Biosciences).
Software	Data collection was done using FACSDiva v8.0.1(BD Biosciences), and data analysis was performed using FlowJo v 10.1
Cell population abundance	Cell are sorted on FACS Canto flow cytometer to >80% purity.
Gating strategy	The green fluorescent protein (GFP)-positive cells were analyzed by flow cytometry (green fluorescence (FITC).

- [x] Tick this box to confirm that a figure exemplifying the gating strategy is provided in the Supplementary Information.
